# Circulating Noncoding RNAs in Pituitary Neuroendocrine Tumors—Two Sides of the Same Coin

**DOI:** 10.3390/ijms23095122

**Published:** 2022-05-04

**Authors:** Henriett Butz

**Affiliations:** 1 Hereditary Tumours Research Group, Hungarian Academy of Sciences-Semmelweis University, H-1089 Budapest, Hungary; butz.henriett@med.semmelweis-univ.hu; 2 Department of Laboratory Medicine, Semmelweis University, H-1089 Budapest, Hungary; 3 Department of Molecular Genetics, National Institute of Oncology, H-1122 Budapest, Hungary

**Keywords:** ncRNA, miRNA, lncRNA, circRNA, pituitary, pituitary adenoma, biomarker, circulating biomarker, liquid biopsy

## Abstract

Pituitary neuroendocrine tumors (PitNET) are common intracranial neoplasms. While in case of hormone secreting tumors pituitary hormone measurements can be used for monitoring the disease, in non-functional tumors there is a need to discover non-invasive biomarkers. Non-coding RNAs (ncRNAs) are popular biomarker candidates due to their stability and tissue specificity. Among ncRNAs, miRNAs, lncRNAs and circRNAs have been investigated the most in pituitary tumor tissues and in circulation. However, it is still not known whether ncRNAs are originated from the pituitary, or whether they are casually involved in the pathophysiology. Additionally, there is strong diversity among different studies reporting ncRNAs in PitNET. Therefore, to provide an overview of the discrepancies between published studies and to uncover the reasons why despite encouraging experimental data application of ncRNAs in clinical routine has not yet taken hold, in this review available data are summarized on circulating ncRNAs in PitNET. The data on circulating miRNAs, lncRNAs and circRNAs are organized according to different PitNET subtypes. Biological (physiological and pathophysiological) factors behind intra- and interindividual variability and technical aspects of detecting these markers, including preanalytical and analytical parameters, sample acquisition (venipuncture) and type, storage, nucleic acid extraction, quantification and normalization, which reveal the two sides of the same coin are discussed.

## 1. Introduction

### 1.1. Pituitary Neuroendocrine Tumors and Diagnostic Challenges

Pituitary neuroendocrine tumors are common intracranial neoplasms with an overall estimated prevalence of 16.7% (14.4% in autopsy studies and 22.5% in radiologic studies) [1]. Although most of them are incidental findings, without significant clinical impact, clinically relevant PitNET are estimated to be found in 0.1% of the general population [1,2]. They can be classified based on different point of views. Clinically, they are usually categorized as functional or non-functional, based on their ability to secrete hormones. Pathologically, they are classified according to the cell type they are originating from, defined using detection of anterior lobe hormone and transcription factors by immunostaining [3]. Radiologic distinction is based on size. Accordingly, microadenomas (<10 mm), macroadenomas (>10 mm) and giant adenomas (>40 mm) can be distinguished. As many of them remains clinically silent for years, they are often discovered when symptoms develop many years after their initial development. They can lead to symptoms due to mass effect (e.g., visual disturbances and dysfunction of the normal pituitary) or through hormone oversecretion. However, even non hormone producing tumors have significant impact on morbidity [4]. Although most of them are benign entities, some (depending on the tumor subtype and the time elapsed between tumor initiation and discovery) can become invasive spreading into the surrounding tissues [5,6]. Determination of invasiveness is also important, as it correlates with prognosis (tumor recurrence) and response to therapy [5,6]. During routine pathological assessment both evaluation of tumor proliferation (mitotic count and Ki-67 index) and invasion are recommended, as these have been demonstrated to correlate with a more aggressive clinical behavior of tumors [3,5,6]. However, in the current WHO classification no specific Ki-67 cut-off value is recommended for diagnosis, and there is no evidence for the utility for p53 immunostaining [3].

Diagnostic and prognostic biomarkers that help early detection when complete surgical removal is possible and aid evaluation of the tumor’s invasive nature should be clinically beneficial. Additionally, while in case of hormone secreting tumors, pituitary hormone measurements usually allow monitoring the disease (e.g., therapy response), in non-functional (NF) tumors the only opportunity for clinical follow up is MR imaging. Therefore, developing non-invasive monitoring biomarkers for NF tumors would be beneficial for clinical practice.

### 1.2. Rationale of ncRNAs as Biomarkers Indicating Pituitary Function and the Potential Presence of PitNET

**Figure 1:**
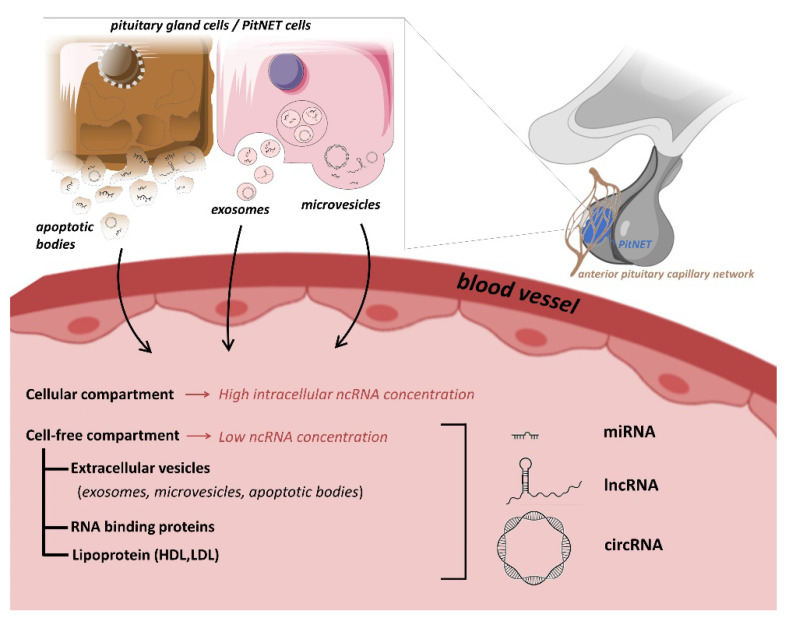
Potential origin and type of ncRNAs as biomarkers in PitNET.

Liquid biopsies (most often blood, urine or saliva samples) represent an easily accessible, non-invasive option for biomarkers. Anatomy and physiology of the pituitary gland also support the theory that blood can be a representative tissue indicating pituitary pathologies: (i) pituitary gland is a hormone secreting organ, (ii) it is highly vascularized and (iii) it is among the areas of brain without a blood-brain barrier [7]. Experimental work also supported this hypothesis. Xenograft studies showed that ncRNAs secreted by xenograft tumors could be detectable in the bloodstream [8,9,10]. In an animal model study, authors investigated exosomal ncRNAs secreted by healthy swine anterior pituitary in vitro. They identified 416 miRNAs, 16232 lncRNAs and 495 circRNAs using high-throughput RNA sequencing [11]. Finally, in an in vivo study monitoring of the tissue-specific global gene expression in humans, authors detected brain-specific transcripts among circulating RNAs [12] (Figure 1).

These findings provide a rationale for the hypothesis that cell-free DNA in the blood can be used to indicate the physiological state of pituitary, and may be diseases of the central nervous system, such as PitNET.

Indeed, liquid biopsies have already been successfully used for the initial diagnosis as well as for prognostic information of pituitary tumors [13].

Measurement of an ideal tumor biomarker must be easy, reliable and cost-effective using an assay that has high analytical sensitivity and specificity. Additionally, based on the recommendations of FDA-NIH Biomarker Working Group it should be specific for a particular disease, able to differentiate between various physiological states, and consistent between different ethnic groups and genders [14,15]. ncRNAs have characteristics that fulfill many of these requirements (see below), therefore they are often proposed as promising candidates for PitNET.

## 2. Diversity, Biogenesis and Function of Non-Coding RNAs

As high-throughput RNA sequencing technologies have become widely available, they revealed that ~90% of the human genome transcribes non-coding RNAs (ncRNAs) [16]. Interestingly, only 1–2% of all the transcripts are derived from protein encoding genes and translated into proteins [17]. Previously, these protein non-coding transcripts were considered as junk or transcriptional noise [18,19,20,21]. Non-coding RNAs are now, however, confirmed to have important roles in cell and RNA homeostasis [18,19,22,23,24].

The family of ncRNA is very diverse with several ways of classification, including according to genomic position, mechanism of action or based on size [25]. Based on size ncRNAs are grouped into small (<200 nt) and long (>200 nt) ncRNAs. Small ncRNAs include microRNAs (miRNA), small nuclear RNAs (snRNA), small nucleolar RNAs (snoRNA), piwi-interacting RNAs (piRNA), transfer RNA (tRNA), small ribosomal RNA (rRNA), and small cytoplasmic RNA (Y RNA). In contrast, lncRNAs have more heterogeneous sizes (from ~200 to tens of thousands of nucleotides) and they are often highly conserved throughout evolution [25]. Similar to mRNAs, they are expressed in a tissue-specific manner [20,26,27]. Another, more recently discovered class of ncRNAs are circular RNAs (circRNAs) that have a covalently closed circular structure. A large number of circRNAs with different lengths and types have been detected in cancer [28].

As ncRNAs have roles in many biological processes, for instance in transcription, splicing, translation, gene expression, cell cycle, imprinting, embryogenesis, development, dysregulated ncRNAs have been implicated in cancer development, with either oncogenic or tumor suppressive properties [25,29,30,31].

### 2.1. Biogenesis and Function of miRNAs

**Figure 2:**
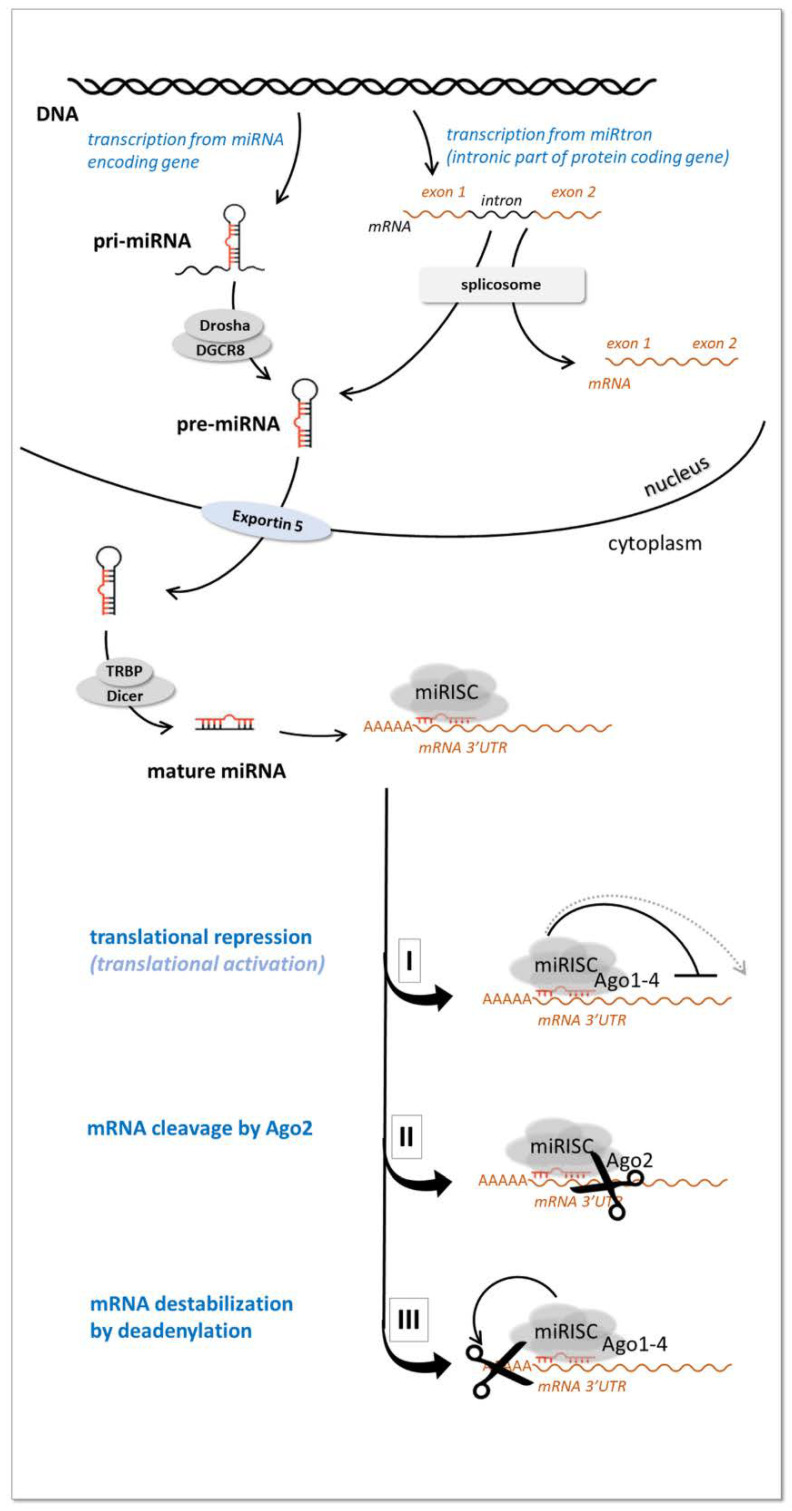
miRNA biogenesis and function (see details in the text).

MiRNAs are the most extensively studied class of ncRNAs [25]. They are short (approx. 19–25 nt) non-coding RNA molecules that posttranscriptionally regulate gene expression via RNA interference by binding the 3′UTR of protein coding mRNAs [32] (Figure 2).

MiRNA encoding genes are localized within introns of protein-coding genes or intergenicly. They can occur individually or clustered through the genome as part of long transcripts encompassing several coordinately expressed miRNAs. After transcribing by RNA polymerase II they undergo a maturation process in the nucleus and in the cytoplasm [33]. In the nucleus primary miRNA transcripts (pri-miRNA) are processed by an RNase III (Drosha) containing complex resulting in a ~60–70 nucleotide long precursor-miRNA (pre-miRNA) characterized by a hairpin secondary structure. The pre-miRNA molecules are then transported to the cytoplasm by Exportin-5, where they are further processed by another RNase III enzyme (Dicer) that cleaves the pre-miRNAs into a ~21 nucleotide long mature (double stranded) miRNAs [33].

One strand of the mature miRNA duplex (guide strand or mature miRNA) is incorporated into miRNA-induced silencing complex (miRISC) while the other strand (passenger strand or “miRNA*” in the previous nomenclature) is usually degraded. In the miRISC complex miRNA interacts with its mRNA target 3′UTR by base-pairing and represses the expression of its targets by three major processes: mRNA cleavage, mRNA degradation by deadenylation or inhibition of translation initiation, Figure 2 (reviewed in Krol et al. [34]). In some particular cases miRNAs can enhance gene expression as well [35]. The role of miRNAs is considered to be mainly to optimize gene expression levels (so called “fine tuning”role) and to ensure adaptive gene expression regulation [36].

It is estimated that approximately 30–50% of all protein-coding genes might be regulated by miRNAs [37]. As one miRNA potentially targets several transcripts, and the expression of each gene can be regulated by numerous miRNAs, the net expressional outcome is the result of the relevant miRNA-target network.

Many miRNAs are present in body fluids (including serum and plasma) and have roles in cell-to-cell communication by functioning as hormone-like molecules to influence the behaviour of different cells in a paracrine or endocrine manner [38,39].

### 2.2. Biogenesis and Function of lncRNAs

In many instances, mRNAs and lncRNAs are more alike than they are different in terms of their biogenesis and structure [40]. Regions encoding lncRNAs include intergenic regions, the sense and antisense orientations of protein-coding genes with transcription start sites located in introns, exons, promoter regions, enhancers, or the 3′- and 5′-UTRs [41]. LncRNAs usually lack an open reading frame, although a few studies have shown their ability to encode proteins [42]. LncRNAs are synthesized by the RNA polymerase II (RNAP II), III complex and spRNAP IV (single-polypeptide nuclear RNA polymerase IV). Similarly to mRNA molecules, the lncRNA transcripts go through RNA maturation and edtiting, such as 5′-capping, 3′-polyadenylation, normal and alternative splicing mechanisms, RNA editing processes, and patterns of transcriptional activation [40,41].

A detailed description of lncRNA’s unique features can be found in excellent reviews [40,41], based on which the following differences from mRNAs can be summarized. In biogenesis, lncRNA genes have a higher enrichment of H3K27ac and are more strongly repressed by certain chromatin remodelling complexes; the transcriptional initiation from divergent promoters differs for the sense (mRNA) and the antisense (lncRNA) directions; the transcriptional elongation is more strongly regulated by DICER1 and MYC for lncRNAs than for mRNAs. Further, lncRNAs are enriched in the nucleus but they can also be localized in the subnuclear domains, the nucleoplasm or the cytoplasm. When cytoplasmic lncRNAs associate with the ribosome few are productively translated. Compared to mRNAs that are degraded in the cytoplasm, lncRNAs can be subject to the nuclear exosome or to cytosolic nonsense-mediated decay (NMD) [40].

**Figure 3:**
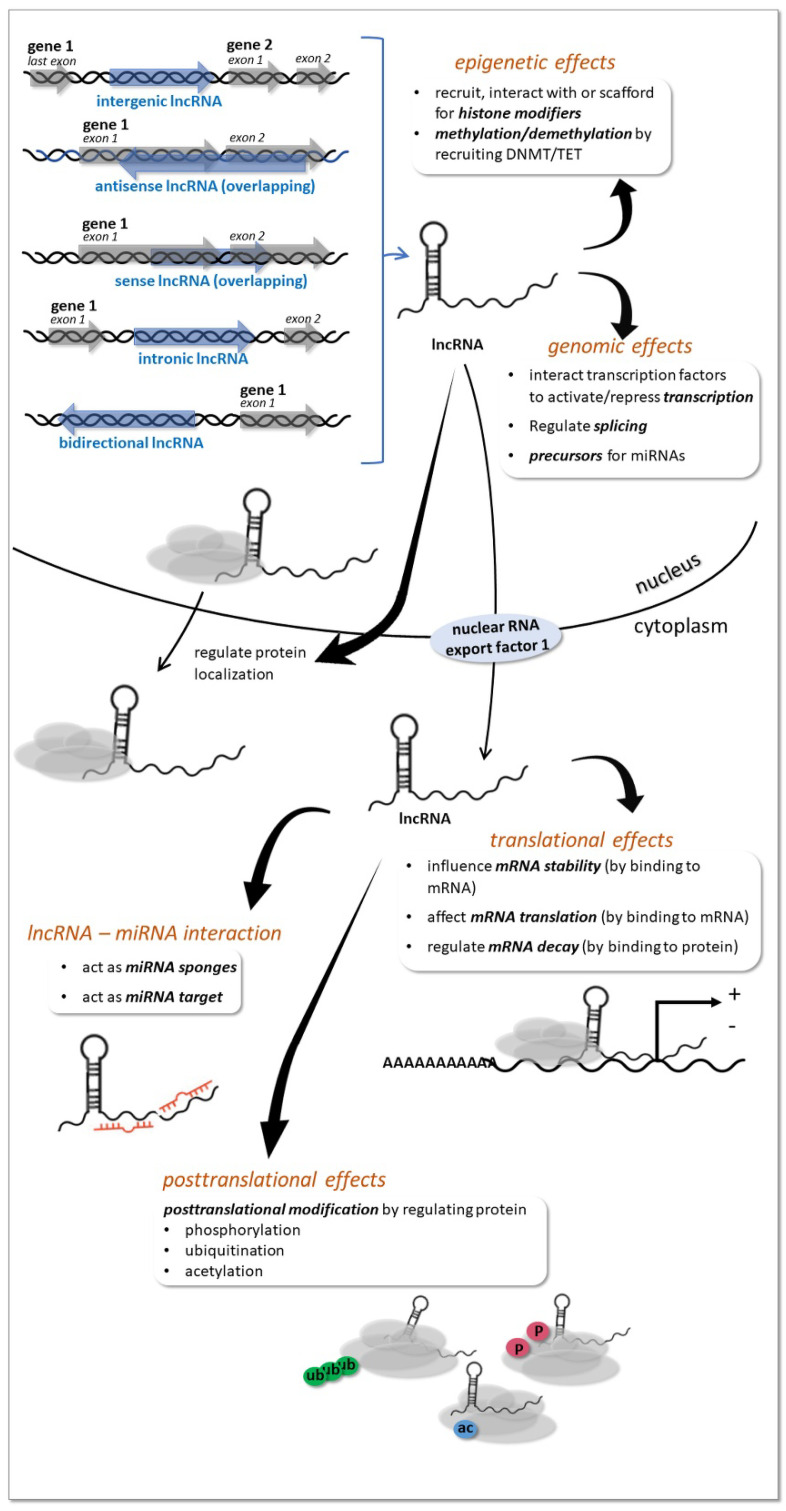
Biogenesis and function of lncRNAs (see details in the text).

LncRNAs are classified based on genomic localization of the encoding region as intergenic (long intergenic ncRNA), intronic (intronic lncRNAs), sense overlapping (sense lncRNAs), antisense overlapping (natural antisense transcripts), and bidirectional [41] (Figure 3). Long intergenic ncRNA may serve as precursors for other types of ncRNA, such as microRNAs. Intronic lncRNAs are generated from spliced introns whose binding to snoRNAs affords subsequent protection from intronic exonucleolytic end-processing mechanisms. Sense lncRNA are exonic lncRNAs that are synthesized from the protein coding portions of genes, therefore their sequence overlaps with their companion mRNAs. Exonic lncRNAs may generated from all of the protein-coding exons, however the majority of them lack functional open reading frames therefore they cannot be translated into proteins. Natural antisense transcripts are transcribed in the opposite direction relative to a protein-coding gene; and may include combinations of exon(s), intron(s), or the full region of the protein-coding genes. Antisense lncRNAs and their respective mRNAs yield pairs of sense-antisense transcripts. Bidirectional lncRNAs consist of transcripts that initiate in close proximity to the start sites of protein-coding genes, and then proceed in the opposite direction. Their characteristic feature is the similarity in expression pattern relative to their companion mRNA. Other, miscellaneous lncRNA types are identified, the description of which exceeds the frame of this work, and the reader can found it elsewhere [40,41].

LncRNAs are involved mostly in RNA homeostasis within the cell, and they can influence gene expression at multiple levels and ways detailed in excellent reviews, specifically, the broad array of functions of lncRNAs include actions on DNA, RNA and proteins, including epigenetic, postrancriptional and postranslational effects [22,30,40,41] (Figure 3). Briefly, lncRNAs can act as epigenetic modifiers, and regulate histone modifications at the chromatin level (epigenetic level). They recruit or interact with histone-modifying enzymes to activate or repress gene transcription. They are also able to act as scaffolds for multiple histone modifiers to regulate histone modification of genes and thereby regulate gene transcription. Further, lncRNAs can also regulate DNA methylation (DNA level).

They are able to recruit DNA methyltransferases/demethylases or are involved in RNA-dependent DNA methylation that regulates gene expression (*transcriptional level*). In addition, lncRNAs are involved in posttranscriptional regulation by acting as miRNA sponges, thereby regulating miRNA-target gene expression and by providing precursors of miRNAs and endogenous siRNAs (*posttranscriptional level*). Additonally, lncRNAs can directly act as targets of miRNAs and can be negatively regulated by miRNAs. miRNAs targeting lncRNAs can produce siRNA or other RNA species, thereby regulating gene expression. Finally, lncRNAs have other roles in posttranscriptional control, such as regulating mRNA decay, mRNA stability, coding polypeptides, protein relocalization, and RNA methylation modification of mRNAs. Beside chromatin, DNA level and posttranscriptional regulation, lncRNAs influence mRNA-protein translation as well. They are able to interact with transcription factors to activate or repress gene expression, and they influence splicing via their interaction with splicing factors or splicing regulating proteins. LncRNAs also affect post-translational protein modification by regulating phosphorylation, ubiquitination and acetylation (*protein level*).

Studies have suggested that some lncRNAs are also present in the extracellular compartments, such as serum, plasma and other bodily fluids in a stable form protected from endogenous RNases [29]. And while there is wider knowledge about the function of lncRNA intracellularly, we have limited information about their extracellular function compared to miRNAs [25,43]. Still, circulating lncRNAs are novel candidates as noninvasive biomarkers in human patient samples [25,29].

### 2.3. Biogenesis and Function of circRNAs

CircRNAs are a class of noncoding RNA species with covalently closed circular structure. A large number of circRNAs with different lengths and types have been detected by high-throughput RNA-seq studies [28]. However, initially, they were often considered aberrant splicing byproducts with little functional potential [44].

**Figure 4:**
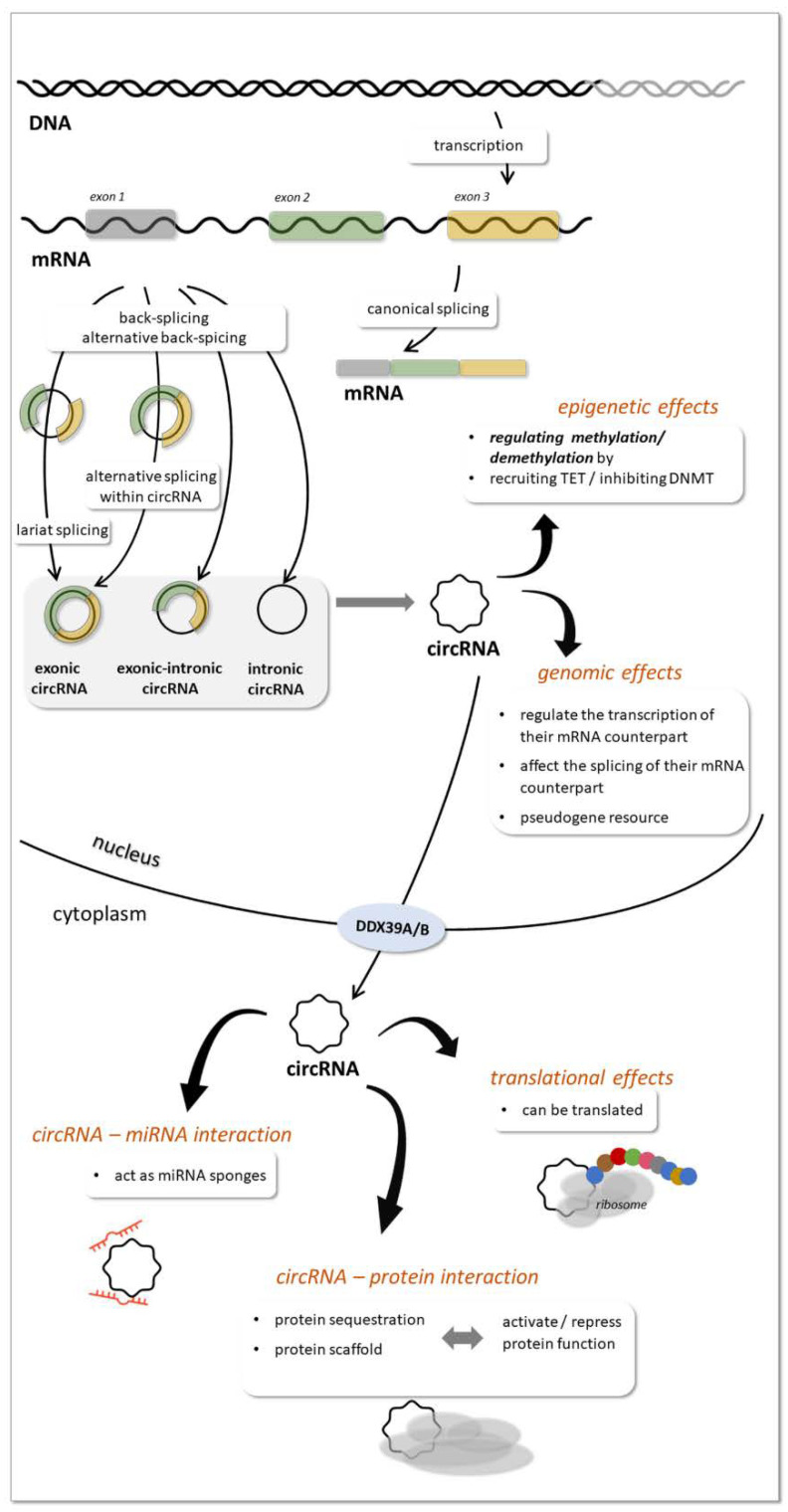
circRNAs biogenesis and function (see details in the text).

A variety of them have been reported to be generated by distinct mechanisms. While the main mechanism of biogenesis is back-splicing of precursor mRNA molecules produced from thousands of genes in eukaryotes, they can also be generated during tRNA and rRNA processing [44]. The circRNAs most studied recently are produced from mRNAs by back-splicing catalyzed by the canonical spliceosomal machinery and modulated by both intronic complementary sequences and RNA binding proteins (RBP) (Figure 4). The different types of circRNAs generated from mRNAs are the exonic, intronic and exonic-intronic circRNAs. Circularization can be lariat driven, when during mRNA splicing 3′ hydroxyl of the upstream exon interacts with the 5′ phosphate of the downstream exon to form a covalent linkage, producing a lariat that contains exons and introns. Further splicing results in exonic circRNA. RBP-driven circularization can also produce exonic circRNAs, while the presence of intronic complementary sequences can lead to base-pairing driven circularization. In this process introns can be removed or retained to form exonic or exonic-intronic circRNA [28,44]. Intronic circRNAs formation requires a 7-nt GU-rich element and an 11-nt C-rich element to escape debranching and exonucleolytic degradation of circRNA [28,44].

Studies also revealed that one gene locus can produce multiple circRNAs with mechanisms related to mRNA alternative back-splicing and alternative splicing within circRNAs [44].

The subcellular compartment where circRNAs are predominantly localized is the cytoplasm [45], although they were also found in the nucleus [46]. Export of circRNAs from the nucleus to the cytoplasm is assisted by DDX39A and DDX39B proteins [47]. In the cytoplasm they can be packed and secreted in exosomes [10] (Figure 4).

In general, circRNAs are expressed at low levels, however, some circRNAs are more abundant than their linear transcripts, and their expression is independent of related linear isoforms [44]. Interestingly, functional studies investigating circRNAs and their linear counterparts proved lower efficiency of back-splicing than the canonical splicing. Overall, the inefficiency of the back-splicing mechanism at most endogenous loci in human cells explains the overall low expression of circRNAs [44].

Due to their unique circular structure that protects them from exonuclease cleavage, circRNAs are considered to be stable. Nevertheless, circRNAs can be degraded, but the exact mechanisms are unclear. Thus far, studies have suggested several possible ways such as degradation by different endonucleases e.g., AGO2 protein, through miRNA sponging or cleavage by RNase P or RNase L [47].

Several mechanisms of action of circRNA are proposed [28,44,47] (Figure 4). Similarly to lncRNAs circRNAs influence gene expression though epigenetic, genomic effects or by intercting with miRNAs or proteins. At the epigenetic level, they can regulate parental genes e.g., by recruiting TET enzymes and inhibititng DNMT1. CircRNAs can regulate transcription of their parental genes by different mechanisms (such as via a positive feedback loop, by promoting Pol II transcription and by binding to DNA and causing exon skipping during mRNA splicing). CircRNAs in the nucleus affect splicing of their linear mRNA counterparts, and in general, the more exons are circularized, the less are processed onto mature mRNA. CircRNAs also compete with the splicing of linear RNAs. In the cytoplasm circRNAs can act as miRNA sponges, thereby reducing miRNA level. By interacting with proteins, circRNAs can activate protein functions by acting as scaffolds or block protein functions by sequestration. Interestingly, a small subset of endogenous circRNAs are translatable or serve as resources for derivation of pseudogenes.

Similarly to other RNA species, the expression patterns of circRNAs are diverse among cell types and tissues and tend to accumulate in cells with a low proliferation rate, such as neurons [44,48]. A significant enrichment of circRNAs was observed in the brain and in cancer development e.g., during epithelial-mesenchymal transition (EMT) [44]. This raises the possibility that they might play significant roles in pituitary tumor development too.

CircRNAs were also reported to be transported by exosomes from the intracellular space to the extracellular fluid [49]. Overall, the secretion of circRNAs in extracellular vesicles (exosomes and microvesicles) was suggested to be a common feature of different cell types [50,51,52]. As in case of miRNAs, the level of circRNAs in exosomes only moderately correlated with that of cellular circRNAs. Hence, it was suggested that the sorting process of circRNAs may be regulated, at least in part, by changes in associated intracellular miRNA levels, and circRNA size may be an additional determinant for selective vesicle export [53,54].

Besides exosomes, circRNAs can be detected as circulating free RNAs (cfRNAs) [55]. Indeed, recent studies have revealed that circRNAs can stably exist in peripheral blood, saliva, urine, gastric juice, seminal plasma and other body fluids due to their circular “closed” structure [44,49]. Although it remains to be determined whether circRNAs could regulate gene expression at distant tissues and cells other than where they are produced, there are initial results confirming their role in cell-cell communication, Specifically, exosomes containing circRNAs retained biological activity by abrogating miRNA, and were shown to induce cell proliferation in vitro [54].

Taken together, the existence of circulating circRNAs suggests that disease-associated circRNAs are promising diagnostic biomarkers [44].

## 3. Non-Coding RNAs as Circulating Biomarkers

The discovery of the presence of ncRNAs in the extracellular space suggested the existence of previously unrecognized novel functions. Indeed, they can be detected in various biological fluids, including serum, plasma, saliva, and urine [43,56]. In addition, different (cellular and cell-free) compartments of the body fluids can contain ncRNAs (Figure 1) [57]. Although extracellular space contains high amounts of RNases, ncRNAs is relatively resistant to nucleolytic degradation as they are protected by being encapsulated [25,58,59]. Extracellular vesicles (EV) (mostly exosomes) were described to be carriers of ncRNAs [25]. However, ncRNAs can also be released from cells in an EV-independent fashion and are detected in complexes with RNA binding proteins, or lipoproteins [55].

Transport mechanisms of ncRNAs to the extracellular compartments involve encapsulation into membranous vesicles (exosomes, microvesicles, and apoptotic bodies) association to RNA-binding proteins (e.g., nucleophosmin, Argonaute protein 2 (Ago2)), or lipoprotein complexes (high-density lipoprotein (HDL)) [43,57]. Exosomes are small vesicles (30–100 nm) that originate from endosomes and are released from cells when multivesicular bodies containing intraluminal vesicles fuse with the plasma membrane [43]. Microvesicles are larger in size (100–1000 nm) and are generated by an outward budding and blebbing process of the plasma membrane [43]. Finally, apoptotic bodies (500–2000 nm) are released from cells undergoing apoptosis. While microvesicles and apoptotic bodies reflect cytoplasmic content, ncRNA profile in exosomes can differ from their parental cell [43,60]. Therefore, it has been hypothesized that a selective sorting mechanism for miRNA packaging into EVs must exist [43]. Sorting can be regulated by specific sequence motifs, posttranscriptional modifications, or subcellular localization [43]. CircRNAs are also detected in exosomes [49] and were found to be about twice more abundant compared to the intracellular compartment of their producer cells [10]. Extracellular vesicles represent a form of intercellular communication, as their ncRNA content can be taken up by recipient cells most probably through membrane receptors [43], and similarly to hormones they act in an autocrine, paracrine, and possibly endocrine manner [61].

Beside EVs, a considerable amount of circulating ncRNAs is associated with ribonucleoproteins or larger complexes [57]. RNA-binding proteins have been identified to be released in association with miRNAs, such as Argonaute 2 (Ago2), that is implicated in the operation of the RNA-induced silencing complex and miRNA-mediated gene silencing, as well as nucleophosmin 1 (NPM1) and nucleolin that regulates nuclear export of the ribosomes [38]. LncRNAs are also detected as part of the cargo of circulating proteins, the origin of these lncRNA-protein complexes and the mechanism by which they are released remains elusive [62]. It was hypothesized that a possible source might be passive leakage of dead or apoptotic cells, and that cells contain potential channels on their cell membrane for selective release of protein-bound ncRNAs [43,62]. In any case, these proteins protect miRNAs from RNase-mediated degradation and potentially stabilize circulating lncRNAs in a similar way [38,62].

Additionally, extracellular miRNAs can be associated with low- and high-density lipoproteins too [57,63,64] (Figure 1).

While there are several studies assessing the function of ncRNAs in cell-to-cell communication, their biological relevance can remain unclear to this date [30,31,39]. Nevertheless, expression patterns of ncRNAs in body fluids have been found to highly correlate with pathophysiological conditions and therefore they are often suggested as next-generation class biomarkers [25,39,62]. Extracellular ncRNAs are stable against temperature, extreme pH changes or repeated freezing-thawing cycles due to their short size (miRNAs), closed structure (circRNA) or their protected nature by EVs [65,66]. Therefore, circulating ncRNAs are good biomarker candidates that have been investigated in several clinical trials, summarized in an excellent review by Anfossi et al. [25].

## 4. Circulating ncRNAs in PitNET

As to date, out of the ncRNA family, miRNAs, lncRNAs and recently circRNAs have been investigated as biomarker candidates in PitNET, therefore these three classes of ncRNAs are discussed below in association with these benign neoplasms. The potential existence of tissue-specific ncRNAs in circulation, their dysregulation in pituitary tumors, their use and the technical challenges are also discussed.

### 4.1. miRNAs as Biomarkers in PitNET

#### 4.1.1. Pituitary Tissue-Specific/Pituitary Function-Specific miRNAs in the Circulation

The theory that pituitary specific ncRNAs might be detectable in the circulation is supported by several lines of evidence [67,68,69,70]. In an in vivo study monitoring the circulating cell-free RNA (mRNA, lncRNA and circRNA) in blood of healthy human individuals, the authors detected transcripts specific to certain tissues, and determined their relative contribution to circulating RNA pool [12]. Interestingly, in this screen brain specific transcripts were identified which were attributed to brain areas outside of the blood-brain barrier. Further, in addition to healthy conditions, upon tissue injury, liver, muscle and brain-specific ncRNAs can be detected in the plasma [67,69,70].

Regarding the pituitary, Hu et al. used miRNA microarray to analyze total RNA from blood samples of 7 children with combined pituitary hormone deficiency (CPHD) and 7 normal controls [71]. In CHPD patients 23 up- and 19 downregulated miRNAs were identified. In the validation phase miR-593 and miR-511 upregulations were confirmed in a larger sample number (103 CHPD vs. 103 healthy control). While the association of the overexpression of circulating miRNAs in a loss-of-function disease with the miRNA origin is intriguing, the identified two miRNAs were able to discriminate the CHPD and healthy controls with high sensitivity and specificity (AUC(miR-593): 0.91, *p* < 0.01; sens: 82.54%, spec: 98.15%; AUC(miR-511): 0.785, *p* < 0.01; sens: 84.86%, spec: 91.36%) [71].

Another study also investigated circulating miRNAs as potential biomarkers for traumatic brain injury (TBI)-induced hypopituitarism [72]. Thirty-eight TBI patients in the acute phase at three different time points and 25 patients in the chronic phase (5 years later) were investigated together with 47 age-gender matched healthy controls. With time, decreasing number of dysregulated miRNAs were detectable in blood (99, 15, 18 and 11 miRNAs at 1, 7, 28 days and 5 years following the injury, respectively). Of the miRNAs identified to be upregulated at different time points only miR-126-3p and miR-3610 were common. These were detected in the sera of patients who eventually developed hypopituitarism on the 1st, 7th, and 28th days, and in the 5th year following TBI [72]. In addition, miRNA-3907 showed statistically significant and constant dynamic changes throughout the investigated time period. While the origin of these miRNAs was not investigated in this study, their presence in circulation was strongly associated with TBI-induced hypopituitarism [72].

The first study investigating pituitary function-related miRNAs in blood was published by Kelly et al. in 2013, who assessed circulating miRNAs as a biomarker of human growth hormone administration to patients [73] (Table 1). Following a microarray screening they selected miRNAs for further validation in 35 plasma samples obtained from individuals with no known pituitary disorders, patients with excess growth hormone (GH) production, and patients receiving therapeutic replacement doses of recombinant human GH (rhGH). As the study of Kelly et al. was designed to investigate miRNAs for detection of doping in athletes, the rhGH replacement group (increased GH) was compared to a combined control group: acromegaly (increased GH)/healthy controls (normal GH). Nevertheless they identifed 4 miRNAs (miR-2861, miR-663, miR-3152, miR-3185) that were downregulated by rhGH administration compared to the control group [73]. Also related to GH function, another study demonstrated that miR-21 containing exosomes derived from human GH producing PitNET tissue promoted bone formation in vitro and trabecula number in vivo [74].

To correlate circulating miRNA with corticotropic cell lineage function, miRNAs in human blood were scrutinized following in vivo administration of dexamethasone and adrenocorticotropin [75] (Table 1). Five miRNAs were selected for the study based on either the fact that they are known to be modulated by ACTH or dexamethasone in an animal model or based on their involvement in the pathogenesis of adrenocortical carcinoma. The levels of these miRNAs were monitored in individuals, who were investigated for dysfunctions of cortisol production. Accordingly, the study included 10 individuals investigated for hypercortisolism (Cushing’s syndrome) using the low-dose dexamethasone test and 10 patients tested for adrenal insufficiency or late onset congenital adrenal hyperplasia (21-hydroxylase deficiency) using the ACTH (tetracosactide) test. The circulating level of miR-27a was upregulated by dexamethasone whereas it was suppressed by adrenocorticotropin in vivo. Secreted miR-27a was also significantly induced by dexamethasone in vitro in NCI-H295R cells. Interestingly, the expression of adrenocortical carcinoma specific miR-483-5p was not affected by dexamethasone or tetracosactide administration [75].

Specific miRNAs were also implicated in the periodical function of hypothalamus-pituitary-ovary axis. Accordingly, the expression pattern of miR-200b and miR-429 in human serum during naturally occurring and induced ovulation cycles was described [76]. The level of miRNA-200b and miRNA-429 is decreased from the early follicular phase toward the early luteal phase. In the mouse knockout model of these two miRNAs, the females were sterile and regained their fertility only after administration of exogenous gonadotropins. Indeed, in anovulatory women with polycystic ovary syndrome, expression of these miRNAs was significantly higher compared with spontaneously ovulating women [76]. In adition, ovulation induction with exogenous gonadotropins reduced their levels to those measured in normal ovulating women [76]. Although this is an intriguing finding, it remains unclear whether this altered miRNA expression profile is a cause or a result of anovulation. The potential pituitary origin of these miRNAs and association with the pituitary gland have also not been investigated. Nevertheless, these findings suggested the hypothesis that these miRNAs act as regulators of hormonal pituitary function.

#### 4.1.2. PitNET-Specific miRNAs in Circulation

The first studies that ivestigated circulating miRNAs in PitNET were related to glioma research. There are three publications where blood samples from patients with PitNET were used as controls besides samples from glioma patients [77,78,79]. As these studies selected miRNAs focused on glioma pathogenesis, the targeted miRNA testing resulted in non significant changes in PitNET samples vs. healthy controls (Table 1).

**Table 1:** Circulating miRNA studies’ characteristics.

Ref.	Patients	Sample Type	RNA Extraction Method	Aim	Detection Method	Endogenous Control	Comparison	Finding	Diagnostic Performance
miRNAs related to corticotrophic PitNET or corticotropine action									
[80]	ACTH-PA (*n* = 28); ectopic ACTH (*n* = 13); HC (*n* = 11)	plasma	miRNeasy Serum/Plasma Kit (Qiagen)	targeted testing (21 miRNAs)	RT-qPCR (TaqMan Advanced MicroRNA Assays)	hsa-miR-191; spike-in control cel-miR-39-3p	ACTH-PA vs. HC	miR-16-5p ↑; miR-7g-5p ↑	na
							ACTH-PA vs. ectopic ACTH	miR-145- 5p ↑; miR-16-5p ↑; miR-7g-5p ↑	AUC(miR-16-5p): 0.879, *p* < 0.001; sens: 90.9%, spec: 77.8%
							ectopic ACTH vs. HC	miR-145- 5p ↓; miR-16-5p ↓	na
[81]	ACTH-PA (*n* = 19); CPA (*n* = 16); HC (*n* = 21)	serum	miRNeasy Serum/Plasma Kit (Qiagen)	whole miRNome	screening: NGS (Illumina TruSeq Small RNA Library Preparation Kit, Illumina HiSeq2500); validation: RT-qPCR (Advanced TaqMan MicroRNA Assays)	hsa-miR-16-5p	ACTH-PA vs. HC	miR-182-5p ↑	AUC(miR-182-5p): 0.87, *p* = 0.0003
							ACTH-PA&CPA vs. HC	no significant miRNA	na
[82] (canine miRNAs)	ACTH-PA (*n* = 19); CPA (*n* = 26); HC (*n* = 6)	serum (HC, CPA); plasma (ACTH-PA)	miRNeasy Serum/Plasma Kit (Qiagen)	targeted testing (20 miRNAs)	RT-qPCR (miRCURY LNA miRNA PCR Assays)	spike-in UniSp2, UniSp4, UniSp5, UniSp6, cel-miR-39-3p, miR-191-5p or the geometric mean of 12 miRNAs	ACTH-PA vs. HC	miR-122-5p ↑, miR-141-3p ↑, miR-222-3p ↑, miR-375-30 3p ↑ and miR-483-3p ↑	na
							ACTH-PA postop vs. preop	miR-122-5p ↓, miR-141-3p ↓	na
							ACTH-PA recurrent (*n* = 3) vs. non-recurrent (*n* = 7) for at least one year after surgery	miR-122-5p ↑, miR-222-3p ↑	na
							CPA vs. HC	miR-483-3p ↑, miR-223-3p ↓	na
[75]	hyCort (*n* = 10), Addison (*n* = 10)	plasma	miRNeasy Serum/Plasma Kit (Qiagen)	targeted testing (5 miRNAs)	RT-qPCR (TaqMan MicroRNA Assays)	spike-in control cel-miR-39	modulated by adrenocorticotropin	miR-27a ↓	na
							modulated by dexamethasone	miR-27a ↑	na
miRNAs related to somatotrophic PitNET or somatotrophic action									
[83]	GH-PA (*n* = 7); NFPA (FSH/LH-PA (*n* = 29); HN/SF1-PA (*n* = 3); HN-Tpit-PA (*n* = 3); plurihorm-PA (*n* = 3)); HC (*n* = 2 + 23)	plasma	miRNeasy Serum/Plasma Kit (Qiagen)	whole miRNome	screening: NGS (QIAseq™ miRNA Library Kit; Illumina MiSeq); validation: RT-qPCR (Advanced TaqMan MicroRNA Assays)	spike-in control cel-miR-39	GH-PA vs. HC	miR-134-5p ↓, miR-152-3p ↓, miR-181a-5p ↓, miR-192-5p ↓, miR-27b-3p ↓, miR-320a ↓, miR-323a-3p ↓, miR-339-3p ↓, miR-378a-3p ↓, miR-382-3p ↓, miR-93-5p ↓, miR-99a-5p ↓	na
							GH-PA postop vs. preop	miR-376a-3p ↑; miR-150-5p ↑; miR-144-5p ↓	na
[84]	GH-PA (*n* = 6), HC (*n* = 6)	serum exosome	PureExo Exosome Isolation Kit; Epicentre Ribo-zeroTM rRNA Removal Kit for ribosomal RNA depletion	whole miRNome	screening: NGS (rRNA-depleted RNA by NEBNext UltraTM Directional RNA Library Prep Kit, Illumina Hiseq); validation: RT-qPCR (miRSCan Panel ChipTM - SYBR based)	spike-in control QB-spike in-1&2	GH-PA vs. HC	miR-320a ↓; miR-423-5p ↓	na
[85]	GH-PA (*n* = 30), HC (*n* = 20)	plasma	Hybrid-RTM miRNA isolation kit (GeneAll Biotechnology, Korea)	targeted testing (miR-29c-3p, miR-31-5p and miR-18a-5p)	RT-qPCR (SYBR-based)	U6 snRNA	GH-PA vs. HC	miR-29c-3p ↓	Association between acromegaly development and downregulation of miR-29c-3p expression: OR (95% Cl) = 1.605 (1.142–2.257), *p* = 0.006
							inadequately controlled (*n* = 7) vs. controlled patients (*n* = not reported)	miR-29c-3p ↓	na
[86]	GH-PA (axcromegaly) (*n* = 47), HC (*n* = 28)	plasma	miRNeasy Serum/Plasma Kit (Qiagen)	whole miRNome	screening: NGS (Illumina TruSeq Small RNA Library Prep Kit; Illumina NextSeq 500); validation: RT-qPCR (Advanced TaqMan MicroRNA Assays)	spike-in control cel-miR-39-3p and miR-191	GH-PA vs. HC	miR-4446-3p ↓; miR-215-5p ↓; miR146a-5p ↓	AUC(miR-4446-3p): 0.862, *p* < 0.001; sens: 89.4%; spec: 82.1%; PPV: 93%; NPV: 83%. AUC(miR-215-5p): 0.829, *p* < 0.001 sens: 78.7%; spec: 89.3%; PPV: 93%; NPV: 91%.
[73]	rhGH (*n* = 6); GH-PA (acromegaly) (*n* = 11); HC (*n* = 3)	plasma	miRNeasy Serum/Plasma Kit (Qiagen)	expression profiling	screening: Affymetrix GeneChipW miRNA 2.0 Arrays; validation: RT-qPCR (SYBR-based)	spike-in control cel-miR-39	rhGH vs. non-rhGH (GH-PA&HC)	miR-663 ↓, miR-2861 ↓, miR-3152 ↓, and miR-3185 ↓	
miRNAs related to NFPA									
[83]	GH-PA (*n* = 7); NFPA (FSH/LH-PA (*n* = 29); HN/SF1-PA (*n* = 3); HN-Tpit-PA (*n* = 3)); plurihorm-PA (*n* = 3)); HC (*n* = 2 + 23)	plasma	miRNeasy Serum/Plasma Kit (Qiagen)	whole miRNome	screening: NGS (QIAseq™ miRNA Library Kit; Illumina MiSeq); validation: RT-qPCR (Advanced TaqMan MicroRNA Assays)	spike-in control cel-miR-39	FSH/LH/HN-PA vs. HC	miR-122-5p ↓, miR-134-5p ↓, miR-152-3p ↓, miR-181a-5p ↓, miR-192-5p ↓, miR-27b-3p ↓, miR-320a ↓, miR-339-3p ↓, miR-378a-3p ↓, miR-382-3p ↓, miR-93-5p ↓, miR-99a-5p ↓	na
							FSH/LH-PA postop vs preop	7 miRNAs (miR-4647 ↑; miR-143-3p ↓; miR-6514-3p ↑; miR-3122 ↑; miR-101-5p ↑; miR-6850-5p ↑; miR-6867-5p ↑)	AUC(miR-143-3p): 0.79, *p* = 0.024; sens: 81.8%, spec: 72.7%
miRNAs related to PitNET (adenoma type mixed or not specified)									
[87]	PA (*n* = 30) (ACTH (*n* = 15), P RL (*n* = 9), GH (*n* = 6)); inv (*n* = 15) vs. non-inv (*n* = 15) (types not defined))	plasma	Trizol	targeted testing (miR-200a)	RT-qPCR (SYBR-based)	U6	inv vs. non-inv (preop)	miR-200a ↑	na
							inv vs. non-inv (postop)	miR-200a ↓	na
							inv preop vs. inv postop	miR-200a ↑	AUC(miR-200a): 0.98, *p* = not reported; sens: not reported, spec: not reported
							non-inv preop vs. non-inv postop	not reported	na
[88]	PA (*n* = 36, types not specified), HC (*n* = 8)	serum	RNAiso Plus	targeted testing (miR-16)	RT-qPCR (SYBR-based)	not reported	PA vs. HC	miR-16 ↓	na
[77]	PA (*n* = 11); glioma (*n* = 66); meningioma (*n* = 32); acoustic neuroma (*n* = 14)	plasma	mirVana PARIS kit (Ambion)	targeted testing (miR-185)	RT-qPCR (SYBR-based)	spike-in control cel-miR-39 and cel-miR-238	HC vs. PA	not significant	na
							HC vs. glioma	miR-185 ↓	na
[79]	PA (*n* = 5); glioma (*n* = 64); HC (*n* = 45); meningioma (*n* = 8); primary diffuse large B-cell lymphoma of the CNS (*n* = 6)	serum	miRNeasy Serum/Plasma Kit (Qiagen)	targeted (miR-205)	RT-qPCR (Advanced TaqMan MicroRNA Assays)	miR-16-5p	glioma vs. PA	miR-205 ↓	na
							HC vs. PA	not significant	na
[78]	PA (*n* = 10); glioma (*n* = 30); meningioma (*n* = 10)	plasma	miRcute miRNA isolation kit (chloroform based)	targeted testing (9 miRNA)	RT-qPCR (SYBR-based)	spike-in control mmu-miR-295	HC vs. PA; HC vs. Meningeioma	not significant	na
							PA vs. glioma	miR-21 ↓, miR-128 ↑ and miR-342-3p ↑	na

Abbreviations: ACTH-PA: ACTH producing PitNET; ectopic ACTH: ectopic ACTH secretion; Addison: patient with Addison syndrome; AUC: area under curve; CNS: central nervous system; FSH/LH-PA: FSH/LH-positive PitNET; GH: growth hormone; GH-PA: GH producing PitNET; HC: healthy control; HN: PitNET with negative staining for anterior pituitary homones; HN/SF1-PA: gonadotrpic PitNET with negative staining for anterior pituitary homones but SF1-positive; HN-Tpit-PA: corticotropic PitNET with negative staining for anterior pituitary homones but Tpit-positive; hyCort: hypercorticolic patient; inv: invasive; na: not available; NFPA: non-functioning PitNET; NPV: negative predictive value; non-inv: non-invasive; PA: PitNET (pituitary adenoma); plurihorm-PA: plurihormonal pituitary PitNET; PPV: positive predictive value; PRL: prolactinoma; preop:preoperative; postop: postoperative; rhGH: recombinant human growth hormone; sens: sensitivity; spec: specificity; SYBR: syber green.

MiR-16 was among the first identified miRNAs with tumor suppressor role in pituitary PitNET [89,90]. These miRNAs were expressed at lower levels in different types of PitNET as compared to normal pituitary tissue. Similarly, the expression of miR-16 in serum samples from pituitary tumor patients was declined compared with the normal group in a targeted testing [88]. The validity of the changes of miR-16 in blood however is sometimes questioned as it is frequently used as endogenous standard due to its stable expression both in tissues and circulation, even in pituitary studies [25,91,92] (Table 1). On the other hand, it is also frequently identified as biomarker in cancer studies [25]. In addition, hemolysis can significantly alter the miR-16 level in blood as it is especially enriched in erythrocytes [25,91,92].

The first comprehensive analysis that used next generation sequencing to profile circulating miRNAs in non-functional (NFPA) and GH secreting PitNET investigated 149 plasma and extracellular vesicle samples (preoperative, early postoperative, and late postoperative) from 45 patients [83]. The authors addressed several aspects of miRNA biomarkers and made key observations as follows. (i) Interestingly, a global downregulation of miRNA expression was observed in plasma samples of patients with PitNET compared with normal samples. (ii) Expression of a set of 29 miRNAs and 11 isomiR in preoperative plasma samples was able to distinguish PitNET histological types and normal plasma samples. (iii) For the identification of potential PitNET-specific miRNAs in circulation, plasma miRNA sequencing data (PitNET vs. normal and pre- vs. postoperative samples) were crossvalidated with miRNA expression profiling studies available in the literature in which normal tissue was used as reference. The authors concluded that differentially expressed miRNAs in PitNET tissues have low abundance in plasma, minimizing their role as biomarkers. (iv) To reveal potential biomarkers that could be used to monitor disease progress, preoperative and late postoperative plasma samples (collected 3 months after pituitary surgery) grouped by different histological types were compared. Three, seven, and 66 differentially expressed miRNAs were identifed between preoperative and late postoperative plasma samples in GH, FSH/LH-positive NFPA, and hormone-negative-NFPA (HN-NFPA) groups, respectively. MiR-143-3p expression in preoperative plasma samples showed significantly higher expression in FSH/LH-positive NFPA gonadotroph samples compared to other histological subtypes. Receiver operating characteristic (ROC) analysis indicated high AUC: 0.79 (*p* = 0.024; sens: 81.8%; spec: 72.7%) in discriminating pre- and postoperative plasma samples (Table 1). (v) To further characterize miR-143-3p, its specificity for FSH/LH-positive NFPA was proved as miR-143-3p level did not change in GH, null cell, SF1-positive-HN NFPA and plurihormonal PitNET in either early or late postoperative samples compared with their preoperative pairs. No association between miR-143-3p expression and PitNET size, Ki-67 proliferation index or FSH/LH ratio was observed. To further investigate potential complicating factors several correlations were assessed. miR-143-3p did not show relevant change in patients undergoing surgery for the first-time vs. patients who previously underwent surgical removal. No significant difference in miR-143-3p level was observed in cases in which complete removal was achieved compared with those in whom a residual/recurrent tumor was detected by imaging. Also, the expression of miR-143-3p in preoperative, early postoperative, and late postoperative plasma samples of patient with posoperative hypothyroidism and hypadrenia showed no differences compared with those of other patients.

Regarding somatortoph PitNET, Zhao et al. assessed miRNA expression profile in exosomes extracted from sera of 6 patients with GH secreting PitNET and 6 healthy controls using two high-throoughput platforms [84]. Two differently expressed miRNAs in GH PitNET samples vs. controls were identified that showed the same direction in expressional change. Both miRNAs (miR-320a and miR-423-5p) showed downregulation in patient samples. Another high-trhoughput miRNA expression profiling cross-sectional case-control study assessed 12 consecutive patients with acromegaly along with 12 age and sex-matched controls [86]. Based on NGS results and validated on an extended sample number, miR-4446-3p (AUC: 0.862; *p* < 0.001) and miR-215-5p (AUC: 0.829; *p* = 0.001) were identified to differentiate patients with acromegaly from healthy controls. In addition, these miRNAs indicated not strong, but significant negative correlation with serum IGF1 levels (miR-4446-3p & IGF1 (τ=−0.34, *p* < 0.001), and miR-215-5p & IGF1 (τ =−0.31, *p* < 0.001)). None of the plasma expression of the two miRNAs correlated with GH or tumor volume [86]. Also in somatotroph PitNET patients, Korkmaz et al. performed targeted measurements of three miRNAs (miR-29c-3p, miR-31-5p and miR-18a-5p) in plasma samples from 20 acromegaly patients and 30 healthy controls [85]. In acromegalic patients miR-29c-3p was significantly downregulated compared to the controls, which was more pronounced in inadequately controlled acromegaly patients than in patients well controlled with somatostatin analogues [85].

Circulating miRNAs were also assessed in corticotroph tumors. High-thoughput sequencing was performed in samples of patients with ACTH-independent and ACTH-dependent Cushing’s syndrome (due to cortisol-producing-adrenal adenoma; CPA or ACTH-secreting PitNET, ACTH-PA, respectively) and in controls where Cushing’s syndrome had been ruled out [81]. Regarding PitNET in the discovery phase, only miR-182-5p was found to be significantly regulated between ACTH-PA samples and controls. However, a set of 6 miRNAs (miR-96-5p, miR-146b-5p, miR-183-5p, miR-185-5p, miR-616-5p, and miR-629-5p) showed pronounced differences between ACTH-PA and CPA. During validation, only miR-182-5p was found to be significantly upregulated in the ACTH-PA group of the studied cohort (AUC(miR-182-5p): 0.87, p = 0.0003) [81]. Another study explored whether miRNAs are able to differentiate patients with ACTH-dependent Cushing’s syndrome (CS) from those with ectopic ACTH secretion (ectopic-ACTH) [80]. For this, 21 miRNAs previously reported to be differentially expressed in ACTH-secreting tumors vs. healthy tissue samples were quantified in plasma by qPCR from 28 subjects with Cushing’s Disease (due to ACTH-secreting PitNET, ACTH-PA), 13 ectopic ACTH secretion (ectopic-ACTH) and 11 healthy controls. While two miRNAs (miR-16-5p and miR-7g-5p) showed increased expression in ACTH-PA plasma samples compared to controls, the levels of 3 miRNAs (miR-145-5p, miR-16-5p, miR-7g-5p) were incresed in samples with ACTH-PA patients compared to ectopic-ACTH syndrome [80]. Moreover miR-16-5p showed high performance in discrimination using receiver-operator characteristics (Table 1). In a canince study, Sanders et al. reported dysregulation of several circulating miRNAs in dogs by comparing serum and plasma samples from dogs with ACTH-PA, CPA and healthy controls [82]. Among the identified miRNAs miR-122-5p, miR-141-3p were overexpressed in samples of dogs with ACTH-PA, and their changes accurately indicated both the successful removel of the tumor and its recurrence. These data suggested that circulating miRNAs have the potential to be non-invasive biomarkers not only in humans but also in dogs.

Independent of PitNET subtype, circulating miRNAs were reported to indicate invasiveness too [87]. Investigation of plasma samples from patients with invasive and non-invasive pituitary PitNET (mixed types), revealed miR-200a to be increased in invasive samples. While the expression of this miRNA decreased in postoperative invasive samples compared to preoperative ones, a similar comparison in non-invasive pre and postoperative samples was not repoted [87]. Nevetheless, miR-200a represents a promising biomarker for diagnosis and potential targets for novel invasive PitNET.

### 4.2. LncRNAs as Biomarkers in PitNET

Compared to miRNAs, lncRNAs relevant to PitNET have been investigated with much less intensity. Nevertheless, expresion and functions of several up and downregulated lncRNAs in PitNET tissues have been explored, as summarized excellenty in: [13,93]. While circulating lncRNAs have emerged as a new class of promising cancer biomarkers [29], they are scarcely investigated in PitNET.

In the recent study by Zhang et al., the authors assessed whether exosomal H19 lncRNA could be transported across the cell membrane to exert its inhibitory effect on pituitary tumor growth [94]. Stable, H19 overexpressed GH3 cell line was used to generate a xenograft model. H19 overexpression inhibited contralateral pituitary tumor growth in vivo. H19 containing exosomes inhibited GH3 proliferation and increased the sensitivity to cabergoline in vitro too. In additon, the expression level of exosomal H19 in the plasma of human patients with all subtypes of pituitary tumors was significantly lower than that in the healthy subjects [94]. Also, the prognostic outcome of medically treated patients with prolactinoma was significantly correlated with the change of exosomal lncRNA H19 level, and dopamine agonist cabergoline increased the expression level of H19, exhibiting a synergic therapeutic effect with exosomal lncRNA H19. Therefore, the authors proposed that plasma exosomal H19 may serve as an important biomarker for predicting medical responses of patients with prolactinomas.

Another study investigated the circulating plasma level of the antisense ncRNA gene at the INK4 locus (ANRIL) in patients diagnosed with PitNET. This ncRNA was chosen, as previous reports demonstrated the oncogenic role of miR-200a and ANRIL in several tumors [87]. In the study plasma samples from invasive and non-invasive mixed types of PitNET patients were used. As in findings in PitNET tissues, the expression of lncRNA ANRIL showed similar pattern to that of miR-200a (see above). In patients with invasive PitNET ANRIL showed significantly higher level than that in patients with non-invasive PitNET before the operation. Additionally, ANRIL expression levels were lower in plasma of patients with invasive PitNET after operation compared to the same patients with invasive PA before operation. ROC analysis indicated that lncRNA ANRIL can distinguish patients with invasive PitNETs between pre- and post-operation period (AUC: 0.79), and invasive PitNET from non-invasive PA (AUC: 0.98) [87].

Another relevant lncRNA in pituitary is Maternally expressed 3 (MEG3), that was found to be dysregulated in clinically non-functioning PitNET [95,96,97]. Its expression is decreased in tumors compared to normal tissue, and even more so in invasive PitNET. This downregulation was associated with the hypermethylation of 14q32 region [95,96,98]. Interestingly, although high plasma MEG3 expression is a good prognostic marker in traumatic brain injury and its circulating level was associated with different tumor types, it has not been investigated in circulation in association with PitNET at all [99,100,101].

### 4.3. circRNAs as Biomarkers in PitNET

Although recent studies have shown that circRNAs may play crucial roles in brain development and diseases, still, they are scarcely investigated in PitNET [93,102].

Indeed, only a few studies have reported the expression and/or function of circRNAs in PitNET. Du et al. identified circOMA1 as a target of miR-145-5p [103]. CircOMA1 (hsa_circRNA_0002316) was demonstrated to sponge miR-145-5p, whose suppression in NFPA cells was abrogated by circOMA1 overexpression. Silencing of circOMA1 caused a similar inhibitory effect than miR-145-5p overexpression. In addition, circOMA1 was shown to upregulate Mcl-1 and Bcl-xL and downregulate Bax (apoptosis-replated genes). Therefore, the authors concluded that circOMA1 promoted NFPA progression [103].

Also in NFPA, circRNA expression profile was compared in invasive and non-invasive tumors [104]. The study authors identified a differentially expressed circRNA signature including 91 upregulated and 61 downregulated circRNAs in invasive tumors. Among there 14 dysregulated circRNAs were suggested to be associated with invasivess. During validation, downregulated hsa_circRNA_102597 significantly correlated with tumor diameter and tumor grade. This circRNA alone or in combination with Ki-67 index was able to accurately differentiate invasive from non-invasive NFPAs as well as predict tumor progression/recurrence [104].

A comprehensive circular RNA profiling in GH secreting PitNET identified thousands of up- and downregulated circRNAs [105]. Using functional assays, the knockdown of hsa_circ_0001368 significantly inhibited proliferation, invasion and GH secreting activity of primary culture cells. Additionally, this circRNA showed significant positive correlation with the pituitary-specific transcription factor Pit-1. Therefore, hsa_circ_0001368 may represent a novel potential biomarker and therapeutic target of GH-producing PitNET [105].

While all these finding are encouraging, data on circRNA in plasma or serum have not yet been assessed in relation with PitNET.

## 5. Potential Causes of Discrepancies in Literature Findings—Technical Aspects

The presence of miRNAs, lncRNAs and circRNAs in blood, together with their association with malignant diseases, provides a rationale for ncRNA-based liquid biopsy [25]. However, reviewing studies assessing circulating ncRNA in PitNET reveals that the dysregulation and role of few (if any) ncRNAs were validated by multiple independent studies. Inconsistencies that exist among the results of different studies may be related to technical aspects.

**Figure 5:**
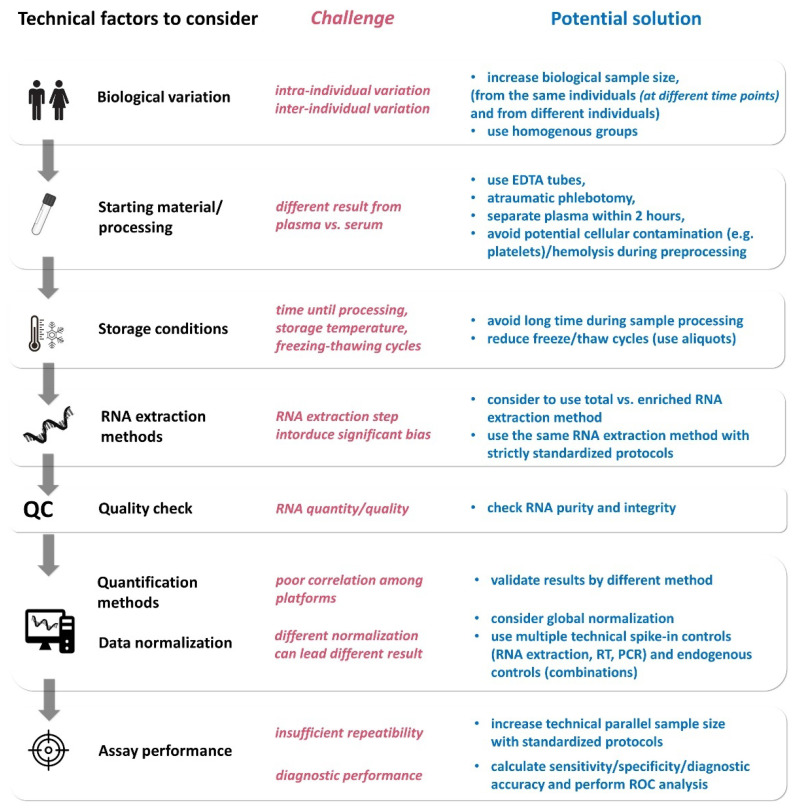
Technical aspects of ncRNA detection (see details in the text).

As for any other routine laboratory tests, the pre-analytical and analytical phases include many variables [106,107], including biological variation and differences in starting material, sample collection, storage conditions, RNA extraction methods, quantity and quality assessment, profiling/measurement methods. Additionally, assay performance also varies, and therefore sensitivity, specificity, intra- and inter-assay variabilities should also be determined. These factors all have to be considered before a certain ncRNA is aimed to be introduced as a clinical laboratory test (Figure 5).

### 5.1. Biological Variation

Even before measurements, and independently of the technical aspects, intra- and interindividual variabilities should be evaluated. A study investigating serum miRNA profiles of matched samples taken from 12 healthy individuals at two different time points (2–17 months depending on the individual) reported a high correlation between miRNA profiles from the same individuals [108]. In another study Ammerlaan *et al.* explored temporal variations of miRNAs over a 1-year period in different blood derivatives (serum, plasma, and specific white blood cell subpopulations), collected every 2–3 months from two healthy donors [109]. The authors identified a continuum of intraindividual temporal variability, with particularly stable (coefficient of variation [CV] < 20–30%) and particularly unstable (CV > 100–130%) miRNAs [109]. Interindividual miRNA variability was also reported [110,111]. Additionally, miRNAs associated with body mass index, sex and age have been also revealed in a study that analyzed plasma samples from 372 individuals after adjustments for technical as well as blood cell parameters [112]. Diet and regular exercise have strong influence on metabolic state that can also manifest in circulating miRNA profile [113,114]. In a study investigating lipids, micro-elements, and vitamins in association with plasma miRNAs revealed that the level of vitamin D, sodium, and vitamin E correlated with the largest number of miRNAs in circulation [115]. Also, circulating miRNA profile in healthy overweight and obese subjects were found to be altered, and the downregulation of miR-361, as a possible biomarker candidate, indicated energy-restricted low-glycemic index diet [116]. Additionally, changes in circulating miR-139 and let-7c were significantly associated with changes in lipid profile and insulin resistance [116]. The effect of diet also demonstrated by the identification of human-specific miRNAs influenced by ketogenic diet [117]. While differences in miRNA expressional changes were found between male and female subjects, three miRNAs, let-7b-5p, miR-143-3p and miR-504-5p were influenced in an equal manner in both sexes after 6 weeks of ketogenic diet [117]. MiRNA changes in association with diet can be an endogenous reflection of physiological changes, they can also be originated from dietary sources representing inter-species RNA transfer. Indeed, bioavailability and distribution of exosomes and their miRNA cargos from bovine, porcine and murine milk within and across species boundaries have been demonstrated [118]. In the study of Manca et al. presented that fluorophore-labeled miRNAs in bovine milk exosomes showed unique distribution profiles and accumulated in intestinal mucosa, spleen, liver, heart or brain following administration to mice [118]. Exercise is another important physiological factor that influence the expression numerous (~380) circulating miRNA level [114,119]. Therefore, it was suggested that long-term exercise may reduce the risk of certain types of cancers among others by regulating plasma miRNA levels [114]. LncRNAs also showed increased inter-individual expression variability compared to mRNA in blood [120]. Regarding circRNAs, inter- and intra-individual diversity was also observed by investigating seven individuals sampled 3 times at one-month intervals. Additionally, results indicated that circRNAs showed less reproducible expression than linear RNAs between different individuals [121].

Time of the blood draw can also be essential because fasting can influence serum/plasma ncRNA content through HDL concentration (significant number of miRNAs can be packaged in HDL) [107]. In addition, a circadian rhythm of serum miRNA was observed in mice, and it is possible that miRNAs undergo a daily variation in humans as well [122].

### 5.2. Sample Type

There is no clear recommendation on whether serum or plasma is more suitable for investigating circulating ncRNAs. Some researchers found strong correlation of ncRNA expression between serum and plasma levels and concluded both serum and plasma would be suitable [8] for miRNA expression studies. Others detected significant differences in miRNA expression in serum samples but failed to observe the same difference in plasma samples of patients compared with controls. Thus, in these studies, overall expression levels in serum did not correlate well with levels measured in plasma [123,124,125]. While Wang et al. observed higher miRNA concentrations in serum samples compared to the corresponding plasma samples, others detected higher miRNA concentrations in plasma compared to serum [111,124,125]. The difference between serum and plasma miRNA concentrations showed associations with miRNA originated from platelets suggesting that the coagulation process affect the spectrum of extracellular miRNA in blood. Therefore, experts suggest caution and consideration of the potential for cellular contamination during preprocessing, that can be countered by removal of subcellular/cellular components by centrifugation. In addition, miRNA release from platelets during coagulation should be considered [91,92,111]. Indeed, perturbations in blood cell counts and hemolysis can alter plasma miRNA biomarker levels by up to 50-fold [92]. Additionally, given that a majority of reported circulating miRNA cancer biomarkers are highly expressed in blood cells as well, results can potentially reflect a blood cell-based phenomenon rather than a cancer-specific origin [92].

Exosomes. Many ncRNAs are highly associated with extracellular vesicles. Methods of extraction, vesicle types and quality (purity, composition) and validation yield intensive discussion and debate among researchers. Regarding this we direct the reader to recommendations made by the International Society for Extracellular Vesicles (ISEV) [126,127]. Nevertheless, the inconsistencies of extraction and validation methods among studies as well as the difficulties of EV-related methodologies makes application of EVs currently not adequate for clinical laboratories.

### 5.3. Blood Collection and Storage

In the clinical routine, EDTA, citrate or heparin are the commonly used anticoagulants for plasma collection [128]. Heparin inhibits the reverse transcriptase and polymerase enzymes used in polymerase chain reaction (PCR) and citrate also interferes with PCR. As next-generation sequencing (NGS) technology utilizes PCR, EDTA tubes should be used for plasma collection when ncRNAs are planned to be assessed [128].

Hemolysis, occurring during venipuncture, or due to a lengthy storage time between blood draw and RNA extraction or freeze-thaw cycles can lead to false results. Cellular lysis should be avoided at all costs as it increases several folds miRNA concentration in the cell-free blood compartment [91,92,111].

Interestingly, it is suggested that miRNA signatures remain stable for at least 24 h in whole blood, while serum or plasma are more prone to time-dependent transcriptomic changes occurring within 24 h [25,129]. Additionally, by McDonald et al. showed that miRNAs were stable at room temperature for 24h and in refrigerated or frozen samples up to 72 h [111]. Freeze-thaw cycles also affect ncRNAs. RNA was shown to be released from residual platelets after freeze–thawing, affecting miRNA signatures. Therefore, separating platelets early and using platelet-free plasma samples are essential for investigating ncRNAs [130]. Although circulating miRNAs in plasma are reportedly detected after up to 8 freeze/thaw cycles without significant changes in expression levels [8], it is becoming increasingly evident that the stability of circulating miRNAs in serum may not be as robust [131,132]. Indeed, prolonged incubation of the sera led to enrichment of vesicle-associated relative to non-vesicle-associated microRNAs indicating that the former might be more stable in serum [131].

As cellular components are releasing miRNAs (and probably other ncRNAs) during the storage period, extracellular ncRNA expression changes after long-term storage, therefore it is recommended that the blood samples should be processed within 6 h, although processing within 2–4 h from collection is optimal [128].

### 5.4. RNA Extraction

Many commercial kits are available for the isolation of ncRNA (miRNAs, lncRNAs, and circRNAs). These are used to isolate total RNA, small RNA or even EV-associated RNAs [25]. Several studies compared the performance of different RNA extraction methods, with different RNA yields or purity [133]. Interestingly, approaches isolating total RNA proved to have better performance compared to kits that enrich small RNA fractions [134]. Others suggested that some kits are better in investigating low abundance miRNAs compared to others [132]. McDonald et al. found that RNA extraction was the least reproducible step, responsible for the highest intraassay imprecision (77–92%), while reverse transcription and qPCR steps contributed minimally (1–6% and 6–17%, respectively) to the overall imprecision [111] (Figure 5). These data emphasize the importance of accurate protocols during RNA extraction.

Depending on the research question, the miRNA quality check is not essential in most cases, because of the low RNA yield from biofluids and many profiling methods are using a fixed volume of total RNA instead of a specific amount of miRNA [107].

### 5.5. Quantification Methods and Data Normalization

While compared to RNA extraction ncRNA quantification introduces lower variability, when high-throughput approaches are applied, the quantification efficiency of each ncRNA molecule should be highly similar. The most frequently used methods for quantification of ncRNAs are reverse transcription followed by quantitative PCR (RT-qPCR) assays, hybridization based microarrays and next-generation sequencing (NGS) [107]. Detailed description of different platforms used for ncRNA expression studies has been provided describing pros and cons and a selection guide for selecting the appropriate platform elsewhere: [25,47,92,135]. Briefly, RT-qPCR based assays are sensitive and provide a fast and easy option to quantify ncRNA, however the throughput can be ranged from small to medium. Digital PCR (dPCR) can provide absolute quantifications and the detection sensitivity is higher than that of RT-qPCR. Hybridization based approached may not have such high sensitivity. Compared to these previous approaches that use specific probes to identify ncRNAs, NGS technology can provide a high-throughput option detecting all ncRNAs, providing the opportunity to discover previously unknown ncRNA molecules. Additionally, NGS can detect sequence and length variability of RNA molecules, such as isomiRs for miRNAs [107].

It is also worth to note that the results can be affected by platform selection, due to the different technical characteristics of various platforms. Usually, strong correlation can be seen in the detected overall expression profile between the NGS and microarray. However, by correlating the differentially expressed miRNAs individually, surprisingly small overlap was found [136]. The authors suggested that this discrepancy may be due to a nonspecific cross-hybridization of miRNA family members or reduced discrimination between unprocessed and mature forms of the miRNAs. Similar results have been recently reported correlating 12 commercially available platforms for analysis of miRNA expression in a study by Mestdagh et al. [137]. Regarding pituitary, Darvasi et al. compared miRNA profile of the same samples investigated by microarray, RT-qPCR based arrays and NGS [138]. The strongest correlation was observed between microarray and TaqMan-array, while the data obtained by NGS were the most discordant even of using various bioinformatics settings. Nevertheless, technical and biological validation showed high correlation (*p* < 0.001; R = 0.96). In another study, Zhao et al. assessed miRNA expression profile in sera of 6 patients with GH secreting PitNET and 6 healthy controls by NGS (Illumina Hiseq X) and PCR array (miRSCan qPCR Panel) methods [84]. While NGS revealed 169 differently expressed miRNAs, PCR array identified only 16 differently expressed miRNAs in GH PitNET samples vs. controls, respectively. Among these, 8 common miRNAs were observed of which only 2 miRNAs showed the same direction in expressional change. These data highlight the challenge of platform selection, and the need of validation for any ncRNA identified through a high throughput test to increase the legitimacy of the findings.

While lncRNAs can be measured using the same methods as mRNA, circRNAs need specific approach. Before quantification, digestion of linear RNAs is needed to distinguish circRNAs from their mRNA counterparts. When NGS is applied, particular attention has to be paid to the method selection. Due to their non-linear conformation and lack of polyadenylated (poly(A)) tails, circRNAs cannot be detected by RNA sequencing using poly(A) enrichment library preparation. Therefore, other library preparation methods such as rRNA depletion or enrichment of circular RNAs should be used. CircRNA enrichment can be achieved using RNase R, which is an enzyme that preferentially digests linear RNAs, and hence, uncovers expression of circRNAs [44].

Due to the circular structure of circRNA, RT-qPCR is susceptible to rolling circle amplification. Therefore, the recommended platform for circRNA quantification is dPCR that can measure absolute copy number with more precision compared to fluorescent in situ hybridization, RT-qPCR or even NGS [47].

Another important issue is data normalization. Use of endogenous control as a valid normalization could help minimize the technical differences and remove systemic bias among different measurements [139]. Depending on the quantification platform, different normalization can be used. For high-throughput data, a global normalization approach is acceptable (normalizing for global fluorescence/total read number), while in case of targeted approaches the use of endogenous control is recommended [107]. An ideal endogenous control should be the same biotype molecule, that is stably and abundantly expressed irrespective of biological variance and medical conditions at a similar range to the target molecule. In the absence of a valid housekeeping normalization, different spike-in controls for input volume can also be applied. The same starting volume for RNA extraction does not guarantee equal amounts of RNA, and the purified RNA yield can differ among samples from different individuals which makes the normalization even more complicated [25]. Still, the application of one or more synthetic spike-in controls during the RNA extraction process can correct the technical variability [140]. Additionally, other spike-in standards can be introduced prior to cDNA synthesis and PCR to control the efficiency of these reactions and normalize results for comparison [141]. In the case of cell-free serum/plasma, considered “low abundance” samples, research practice often utilizes fixed volume of samples (e.g., 20 µL RNA). In contrast, in the case of high RNA content samples fixed RNA amount (e.g., 1 µg total RNA) is used as starting material for quantification. When using low abundance samples such as liquid biopsy samples, absolute quantification can also be applied, using standard curves generated by serial dilution of synthetic oligonucleotide standards [141]. However, most studies apply relative quantification selecting endogenous controls. The issue of endogenous controls cannot be overemphasized, as the selection of endogenous control can significantly affect results, especially in case of circulating ncRNAs [142]. The problems associated with endogenous controls and data normalization are further discussed in detail by Anfossi et al. [25].

All these technical aspects highlight the need for standardization of sample collection and preparation for blood-based studies of ncRNA. In fact, there are already efforts to generate harmonized/standardized protocols, although every laboratory should validate their own approach [143].

## 6. Conclusions

The development of serum/plasma biomarkers is of great interest for clinical practice as it offers a relatively non-invasive sample withdrawal procedure, compared to tissue biopsy (in case of PitNET: surgery), and fast and cost-effective analysis.

Regarding the pituitary, in spite of existing hormonal biomarkers, there is still a need for biomarkers of PitNET, especially for non-functioning tumors, where the diagnosis and follow-up currently mostly relies on imaging. As ncRNAs are an emerging class of biomarker candidates, their potential use in the near future cannot be disregarded. While many encouraging research studies on circulating ncRNA have been published, ncRNAs has not been widely introduced in clinical routine in patients with PitNET or other tumor types. Several potential reasons might be account for this delay. (i) From a biological point of view, although it is an attractive theory that miRNAs can be secreted from pituitary tumor, research data indicated that even if this is true, they have low abundance in plasma, minimizing their role as biomarkers. (ii) While in adrenocortical carcinoma tumor-specific miR-483-5p was consistently detected by several studies in both serum and plasma, in PitNET no such miRNA has been identified to date. Moreover, comparing results of independent studies, common dysregulated miRNAs are scarcely identified. While this phenomenon is present also in case of other tumor types, it is not encouraging. (iii) Discrepancies are often explained by both biological and technical variables. For implementation of a novel laboratory test to routine clinical practice several factors have to be addressed. From a biological point of view, intra- and interindividual variability have to be assessed in order to establish a base line or reference range. Numerous other physiological (age, gender, fasting status, ethnicity, diet, circadian rhythm, hormonal period etc.) and pathophysiological factors (confounding other diseases such as diabetes, hypertension etc. or neoplasms) have to be investigated to establish specificity. From a technical point of view, numerous preanalytical and analytical parameters from venipuncture, sample type, storage, nucleic acid extraction, to quantification and normalization should be optimized and standardized. Special attention needs to be given to avoid cellular contamination and cellular lysis during the preanalytical phase as ncRNAs exist at many folds higher level intracellularly compared to the cell-free blood compartment. From a research point of view, differences in study design (different tumor type, sample size and technical methods) might also explain the discordant results. Validation by independent groups and multicenter efforts can easily bridge these challenges. There are expert recommendations and guidelines for the introduction of a new biomarker (ERDN, FDA) that should be considered during development.

In summary, the implementation of the use of circulating ncRNAs as biomarkers is currently not feasible in clinical laboratories due to the abovementioned reasons. Nevertheless, we cannot ignore the novel experimental findings, and likely advances in technology and protocol harmonization will lead us to the discovery and clinical implementation of novel ncRNA biomarkers in PitNET.

## Data Availability

Not applicable. All data are included in the manuscript.
